# Characterizing the maximum number of layers in chemically exfoliated graphene

**DOI:** 10.1038/s41598-019-55784-6

**Published:** 2019-12-20

**Authors:** Péter Szirmai, Bence G. Márkus, Julio C. Chacón-Torres, Philipp Eckerlein, Konstantin Edelthalhammer, Jan M. Englert, Udo Mundloch, Andreas Hirsch, Frank Hauke, Bálint Náfrádi, László Forró, Christian Kramberger, Thomas Pichler, Ferenc Simon

**Affiliations:** 1 Faculty of Physics, University of Vienna, Strudlhofgasse 4., Vienna, A-1090 Austria; 2 Department of Physics, Budapest University of Technology and Economics and MTA-BME Lendület Spintronics Research Group (PROSPIN), PO Box 91, H-1521 Budapest, Hungary; 3 Laboratory of Physics of Complex Matter, École Polytechnique Fédérale de Lausanne, Lausanne, CH-1015 Switzerland; 4 Yachay Tech University, School of Physical Sciences and Nanotechnology, 100119 Urcuquí, Ecuador; 5 Institut für Experimental Physik, Freie Universität Berlin, Arnimallee 14, 14195 Berlin, Germany; 6 Department of Chemistry and Pharmacy and Joint Institute of Advanced Materials and Processes (ZMP), Friedrich-Alexander University of Erlangen-Nürnberg, Nikolaus-Fiebiger-Str. 10, 91058 Erlangen, Germany

**Keywords:** Optical properties and devices, Electronic properties and materials, Electronic properties and devices

## Abstract

An efficient route to synthesize macroscopic amounts of graphene is highly desired and bulk characterization of such samples, in terms of the number of layers, is equally important. We present a Raman spectroscopy-based method to determine the typical upper limit of the number of graphene layers in chemically exfoliated graphene. We utilize a controlled vapour-phase potassium intercalation technique and identify a lightly doped stage, where the Raman modes of undoped and doped few-layer graphene flakes coexist. The spectra can be unambiguously distinguished from alkali doped graphite, and modeling with the typical upper limit of the layers yields an upper limit of flake thickness of five layers with a significant single-layer graphene content. Complementary statistical AFM measurements on individual few-layer graphene flakes find a consistent distribution of the layer numbers.

## Introduction

Graphene, the latest discovered carbon allotrope^1,2^, holds promise for a wide range of potential applications from medical devices to sensors^3–6^. Apart from individual graphene flakes, bulk graphene is exploited in numerous systems^7,8^. Notably, bulk single-layer and few-layer graphene (SLG and FLG) are proposed as applicable in efficient Li-ion batteries, components of photovoltaic cells, and are viable candidates for spintronics applications^7,9,10^. Thus, scalable methods are required both for high-yield production and characterization techniques. One of the major challenges, i.e., to establish mass-production techniques leading to high-quality SLG and FLG was overcome in recent years^11,12^. Amongst the numerous synthesis (mostly top-down) means towards mass graphene production, wet chemical exfoliation methods prevail in terms of material quality and synthesis facility^13–16^. Nevertheless, measurement methods suitable for large sample quantities are still lacking.

Raman spectroscopy evolved to be an essential probe for studies of carbon structures especially of nanocarbon^17–19^. It was demonstrated to be an ideal characterization tool not only in the laboratory but also at the mass-production level. In particular, investigations of single-layer graphene are facilitated by the lack of a band-gap in the band structure leading to resonant processes at all wavelengths^20^. Details of the Raman spectra can reveal the layer number of single graphene flakes or identify the edge of the flakes^21–24^, it also enables detailed studies of electrostatic gating, strain, or chemical modifications of graphene^25–36^.

Historically, Raman, phase contrast spectroscopy and AFM studies focused on the characterization of separated graphene flakes^17,37,38^. Bulk analysis of the number of the layers of unmodified bulk graphene is not feasible by optical means. Raman spectra of SLG and FLG single flakes differ significantly in their 2D mode (the overtone of the defect-induced D mode), which provides a way to identify the number of graphene layers, *N*^39^. However, twisted multilayers might have 2D modes resembling SLG^20^. Furthermore, the methods reviewed in Ref. 20 utilize the layer number dependence of the interlayer shear mode^40^, i.e. the so-called C peak and the interlayer breathing modes^41–43^, the ZO’ modes. However, this cannot be applied for powder samples built up of a distribution of different thicknesses.

Here, we present a compelling analysis method based on Raman spectroscopy for analyzing the typical upper limit of the number of graphene layers in a powder sample. We utilize controlled vapour-phase potassium intercalation to distinguish SLG and FLG content through following a stepwise intercalation process. This technique enables us to track the evolution of doping in lightly doped stages, where the Raman modes of the undoped and the doped FLG flakes coexist. The applicability of this intercalation method is demonstrated on a few-layer restacked graphene, poly-dispersed powder prepared by wet chemical exfoliation. The method reveals that the studied samples are composed of graphene flakes with dominantly less than five layers. AFM statistical measurements on individual SLG and FLG flakes of the same material confirm the observed distribution.

## Results and Discussion

**Figure 1:**
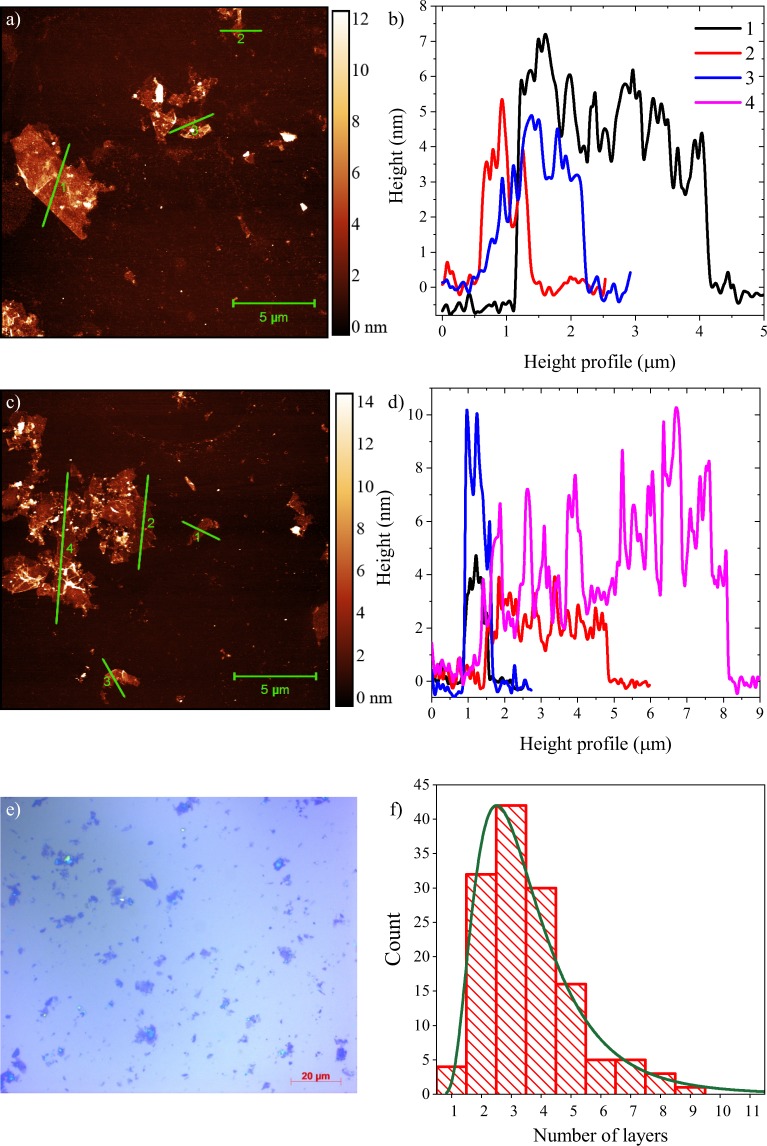
AFM experiments on chemically exfoliated few-layer graphene made with DMSO and ultrasound treatment. Multiple characteristic types of flakes can be identified: (**a,c**) show AFM studies containing few layer graphene sheets (up to 5 layers). Note the diverse lateral size of the flakes that shows that these are partially restacked on the substrate. (**b,d**) Height profile of corresponding graphene flakes along the lines indicated in the left images. (**e**) Light microscope image depicting the distribution of flakes on a 100 × 100 *μ*m surface. (**f**) Distribution of flakes as a function of layer number in the AFM statistical analysis. Solid green line is a lognormal distribution fit to the height profiles revealing a mean of 3 layers for the thickness of flakes. Note that the height of each graphene layer is measured here by AFM to be 1.2 nm. We used the so-called step height analysis method as described in Ref. 44,68.

We performed a detailed AFM statistical analysis study on a large number of as-prepared ultrasound treated individual FLG flakes in a single batch chosen randomly. Figure 1e shows a light microscope image on a 100 × 100 *μ*m surface, revealing a distribution of flakes on the surface. Representative AFM images of the graphene flakes are presented in Fig. 1 along with cross-sectional cuts of the flakes. Figure 1a–d point to presence of graphene flakes with up to five layers, with a sizeable fraction of mono-layer flakes.

Figure 1e shows the distribution of flake thicknesses in our statistical analysis. This analysis highlights that 90% of the chemically exfoliated flakes are composed of maximum 5 graphene layers. A simple fit to a lognormal distribution points to a distribution of flakes centered at 3 layers (with a variance of 1.5 layers). Relevantly, a fraction of the flakes consists of single-layer graphene flakes in our sample. Note that the AFM statistical analysis is only presented for the ultrasound-treated FLG flakes.

Although AFM-based thickness measurement of individual flakes is a standard method for graphene characterization, it was suggested^11,44,45^ that this approach may be misleading due to the improperly chosen measurement parameters, and complementary studies are required. In particular, partial restacking of the chemically exfoliated graphene flakes on the substrate may lead to bigger aggregates of multiple layers where the graphene sheets are misaligned. AFM, however, is unable to resolve this change of the flake morphology, and cannot identify the thickness of individual flakes.

This diversity of the flakes highlights the need for bulk characterization methods, such as Raman spectroscopy. Micro-Raman spectroscopy in our case has about a 5 … 10 *μ*m lateral and vertical resolution, which is large enough for a representative surface average without the biasing effects of nano-imaging.

**Figure 2:**
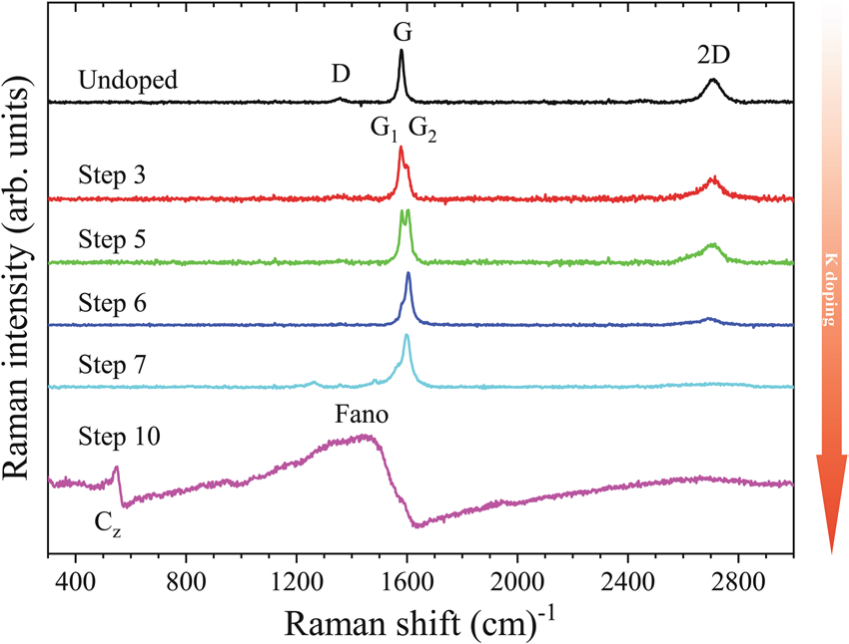
Raman spectra of *in-situ* potassium doped FLG starting from the undoped material (top) towards saturation doping (bottom). Saturation intercalation is reached after about 10 intercalation steps, which are described in the text. Note that several steps are skipped in the figure that show little or no change. Upon doping, the D mode quickly disappears in accordance with previous literature data^50^. The 2D mode acquires some structure but also disappears after further intercalation steps. The G-band splits into G_1_ and G_2_, whose origin is discussed in the text. In the final, fully intercalated step, the G bands form a Fano-shaped band and a C_z_-mode is observed at wavenumbers ~560 cm^−1^, similarly to Stage I graphite (KC_8_).

Starting from undoped FLG, we performed controlled temperature-gradient driven potassium doping experiments. Saturation doping was achieved in approximately 10 steps. We intentionally refer to “steps” in our experiments rather than “stages”, as the latter is reserved for the well-known intercalation stages of bulk graphite^46^. The corresponding Raman spectra (recorded at 514 nm) are depicted in Fig. 2. Raman spectra of the starting material display the usual D, G, and 2D bands and it reproduces the earlier report on similar samples^33^. The 2D band of the starting material is best fitted with a single Lorentzian line, unlike the composed structure in graphite. Whereas the width of the Lorentzian hints that the material may be a mixture of flakes with different number of layers, the Raman response of the starting material is insufficient to determine the exact distribution of the thicknesses. Here, it is worth to note that FLG flakes are stable on their own (i.e. self/standing), no substrate was used during the Raman measurements.

Upon light potassium doping, the Raman spectrum changes significantly: the weak D-band rapidly disappears and the G- and 2D-modes split. At higher doping levels, the intensity of the double-resonant 2D peak components is suppressed, and both signals downshift. The relatively rapid disappearance of the 2D mode hinders its use for further qualitative analysis thus we focus on the G mode and its vicinity. During the first intercalation steps (Steps 1–5), we used each time twice longer intercalation times than at the previous Step. In the meantime, we kept a constant and large temperature gradient to homogenize the material at a small and fixed doping level. Comparison of the *inhomogeneous* Step 3 and the *homogeneous* Step 5 reveals that only the intensity ratio of the G modes changes, the position of the 2D modes is unchanged. Step 5 is of particular importance, as this was found to be a stable phase. The highest doping level (Step 10 in Fig. 2) leads to a radical change of the Raman spectrum. A Fano-shaped line^47,48^, centered around 1486 cm^−1^, and a so-called C_z_-like mode dominate the spectrum^49^. The Fano shape is a clear sign of significant charge transfer to the graphene sheets, which leads to a quantum interference of the zone-centre phonons and the electronic transitions.

It is intriguing to compare this spectrum with the Raman spectrum of Stage I potassium intercalated graphite (KC_8_), where similar Raman bands appear upon intercalation. A detailed analysis is given in the Supplementary Materials, and it indicates that the position, the width (Γ_Fano_), and the coupling strength of the electronic continuum, measured by the asymmetry parameter, *q*, differ. The C_z_ mode arises from from a vibration, where carbon atoms displace perpendicularly to the graphene sheets. Even though this mode exists at the **M** point of the Brillouin-zone, it is folded back to the Γ point due to a 2 × 2 doubling of the unit cell during intercalation^46,49,50^. The presence of the C_z_ mode is a clear indication of a 2 × 2 ordered potassium lattice present on the graphene sheets, or between the graphene layers^46,49,50^. As a consequence, this mode is naturally present in Stage I KC_8_ and is a clear indication of successful and high doping yield.

The most surprising observation in Fig. 2 is the presence of a doublet G mode. In a sample, which contains SLG only, homogeneous doping is expected to lead to a single G mode only. We can rule out the presence of inhomogeneous doping^50^ as we studied a large number of positions on the sample, several intercalation runs, and the same spectra were observed in all cases. It is worth noting, that inhomogeneously doped graphene has indeed a doublet structure, where the Fano line is intermixed with the upshifted G mode^50^. We wish to point out that in our case the origin of the doublet structure is not an inhomogeneous doping but the fact that within the about 1 *μ*m^2^ of the microscocope spot, graphene with varying numbers of layers is present. It is however intriguing that the Raman spectrum of normal bulk graphite shows similar doublet structure under doping. In particular, the Raman spectrum of our Step 5 intercalated FLG may appear similar to a high stage (KC_72_ or Stage VI) GIC. Nevertheless, the spectroscopic details are markedly different.

**Figure 3:**
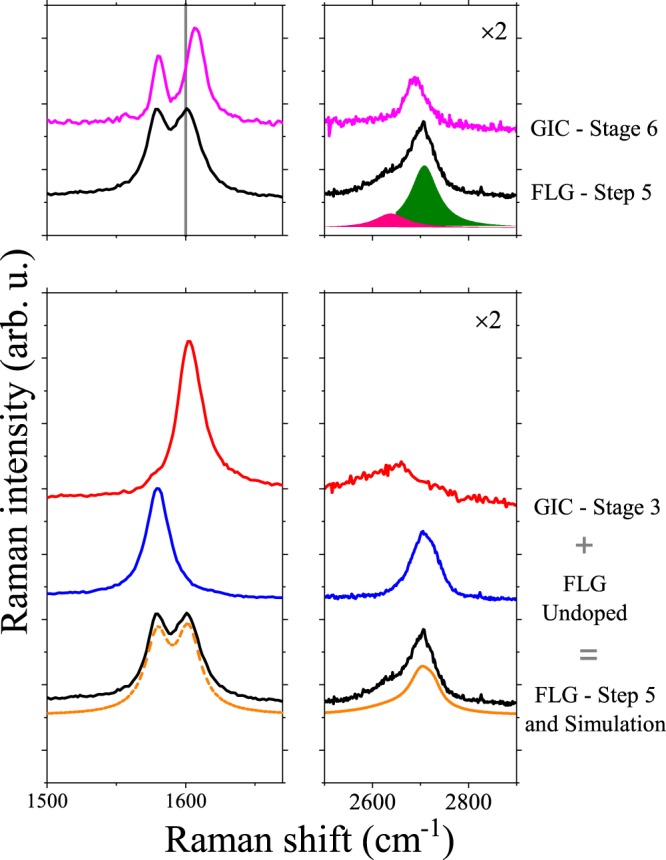
Upper panel: Comparison of single-crystal graphite doped to Stage 6 and FLG doped to Step 5. The vertical line indicates the position of G_2_ line in the doped FLG. A fit with two components (green and pink) simulates well the doped FLG signal. Lower panel: Simulation of the decomposition of the Raman spectrum of FLG doped to Step 5 as a mixture of a stage 3 GIC doped and the undoped FLG material. The bottommost spectrum is the simulated curve shown together with the Step 5 intercalated FLG (thus shown twice in the figure for clarity).

In Fig. 3., we compare the Raman spectrum of our Step 5 intercalated FLG with the data on a graphite single crystal at a doping stage of 6 or KC_72_. Although the doublet structure of the G mode appear similar, two details are different: (i) the G mode with the larger Raman shift lies with about a 6(1) cm^−1^ difference in the two types of materials, (ii) the 2D mode is markedly different in the two kinds of materials: the FLG contains a 2D mode component with a smaller Raman shift, which is absent in graphite. Albeit these difference may appear to be subtle, these enable us to qualitatively differentiate between the two types of materials. The figure also shows that Step 5 intercalated FLG can be resolved into a mixture of a Stage 3 GIC and the undoped material. This fitting procedure (see Supplementary Material) is capable of explaining both the position of the G mode and the composite structure of the 2D mode. This indicates an interesting scenario for the Raman spectrum of the alkali intercalated FLG: it consists of a mixture of (1) entirely undoped pieces, whose Raman spectrum remains identical to that of the starting material, and (2) relatively highly doped phases (equivalent to Stage 3 GIC).

We emphasize that the origin of the doublet structure in GIC is related to the presence of charged and uncharged graphene layers; graphene layers in GIC, which are adjacent to an alkali layer are charged, whereas two which are further apart, remain neutral or uncharged^51^. In this respect, charges in GIC and our stepwise doped FLG are both inhomogeneously distributed, however, the inhomogeneity is completely different. Intercalation in graphite proceeds from homogeneous and crystalline graphite and the charging inhomogeneity is due to the intercalation itself: it occurs due to the thermodynamic preference for fully doped alkali layers which are inevitably separated by uncharged graphene layers. However in our FLG material, the inhomogeneity is present *a priori* in the sample (in terms of the different layer numbers in the grains) and the inhomogeneous doping merely reflects this inhomogeneity as we show below.

**Figure 4:**
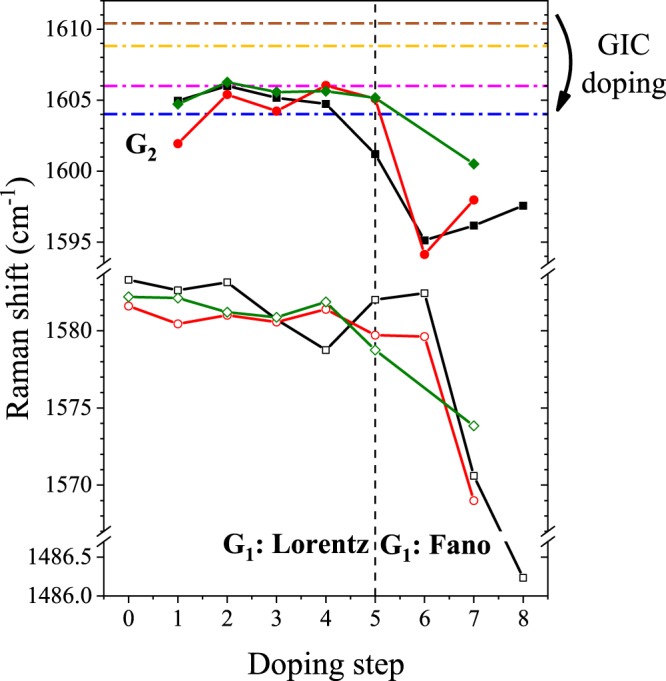
Position of the G_1_ (open symbols) and G_2_ (filled symbols) Raman modes as a function of the doping step in the investigated FLG species at 514 nm laser wavelength. The ultrasound treated material is shown with black, the shear mixed one is represented with red and the mechanically stirred sample with green color. The 0th doping step corresponds to the starting materials. Positions are obtained through fitting the peaks with Lorentzian and Breit-Wigner-Fano functions, transition between the two shapes is denoted with a vertical dashed line. Relevant G_c_ modes of the potassium intercalated GICs are shown with dashed-dotted lines: KC_24_ (blue), KC_36_ (magenta), KC_48_ (yellow), KC_60_ (mahogany)^51^. The error of the measurement is represented with the size of the used symbols.

To gain deeper insight into the composition of the multiple restacked FLG, we analyze the G- and 2D Raman-bands. Intercalation step dependence of the split G-bands (G_1_ around 1580 cm^−1^ and G_2_ around 1600 cm^−1^) are shown in Fig. 4 for all three investigated types of samples. To understand the origin of each G band, we recall the Raman response properties of potassium doped GICs. Therein, the upper and lower G lines were attributed to charged (the G_c_ band) and uncharged layers (the G_uc_ band), respectively^51^. Upon doping, the G_c_ band moves to lower Raman shift beyond experimental error (horizontal lines in Fig. 4). The charges transferred from potassium accumulate on the layer immediately adjacent to the potassium layers, which give rise to the G_c_ band. Charge transfer to the rest of the layers (the so-called inner layers) remains low and varies with stage numbers of the GIC. We note that the G_uc_ band also shifts slightly between the different stages due to strain effect.

In FLG, the G_2_ band arises from charged graphene layers. However, the comparison with the position of the different GIC stages (see Fig. 4) unveils a markedly different behavior for the G_2_ band in FLG and the G_c_ band in GIC. Namely, the position of the G_2_ band is i) independent of the doping steps, ii) its Raman shift position lies between the position of charged G-band in KC_24_ and in KC_36_. This is a strong indication that the G_2_ band corresponds to graphene layers that appear to be doped as in Stage 2 or 3 graphite. It also means that in our FLG samples, no higher stages (or lower doping levels) can be achieved. Given the heterogeneous nature of the number of layers in the FLG sample, this reveals that our sample is free from flakes with more than 3–5 restacked graphene layers. This observation is in full agreement with our AFM statistical analysis.

Figure 4 shows that the position of the G_1_-line barely changes as a function of doping as long as a Lorentzian line fits best the Raman line. This exposes that the induced strain is not affected by the doping level, hence, the G_1_ line corresponds to a significant amount of undoped flakes. Presence of these undoped flakes along with appearance of Stage 3 doping in five-layer-thick flakes highlights the important contribution of undoped flakes with smaller thickness (mono-, bi-, tri-, and four-layer ones).

To further emphasize the differences between the FLG and the graphite powder, we extract a measure of the charge transfer, the electron-phonon coupling parameter (EPC). The electron-phonon scattering linewidth can be estimated from the positions of the Fano lineshape using the expression

1 $${\gamma }^{{\rm{EPC}}}=2\sqrt{({\omega }_{{\rm{Fano}}}-{\omega }_{{\rm{A}}})({\omega }_{{\rm{NA}}}-{\omega }_{{\rm{Fano}}})}.$$

Here, *ω*_Fano_ is the measured position of the G-line peak, *ω*_A_ and *ω*_NA_ are the calculated adiabatic and non-adiabatic phonon frequencies^49,52^. We approximate the latter two quantities with the ones calculated for KC_8_: *ω*_A_ = 1223 cm^−1^ and *ω*_NA_ = 1534 cm^−1^, as no exact calculation exists for FLG. This approximation was found to be valid in similar hexagonal carbon systems such as potassium doped multiwalled carbon nanotubes^53^.

The extracted values are summarized in Table 1. Therein, Γ_Fano_ is the linewidth of the Fano lineshape. In accordance with previous findings in GICs^52^, the *γ*^EPC^ of SGN18 and FLG follow the linewidth of the Fano lineshape linearly (${\Gamma }_{Fano}\approx {\gamma }^{EPC}$). Comparison of the measured characteristics reveals that charge transfer is the largest in FLG and in HOPG, followed by SGN18. Weaker charge transfer in SGN18 can be explained by its morphology, as powders are more difficult to intercalate^54^. Thus, the larger charge transfer in FLG in powder form is a remarkable proof of a system with weak internal strain due to the majority of one- to three-layer flakes.

**Table 1:** Electron-phonon coupling parameters from the analysis of the G-modes.

Sample	*ω*_Fano_	Γ_Fano_	*q*	*γ*^EPC^
FLG step 10	1505	148	−1.5	181
SGN18 Stage I	1515	89	−0.7	148
HOPG Stage I (Ref. 49)	1510	118	−1.9	166

The values of *ω*_Fano_, Γ_Fano_, and *γ*^EPC^ are in cm^−1^. Calculated parameters in maximally intercalated FLG are compared to values found in graphite powder (SGN18 Stage I), and Stage I HOPG.

**Figure 5:**
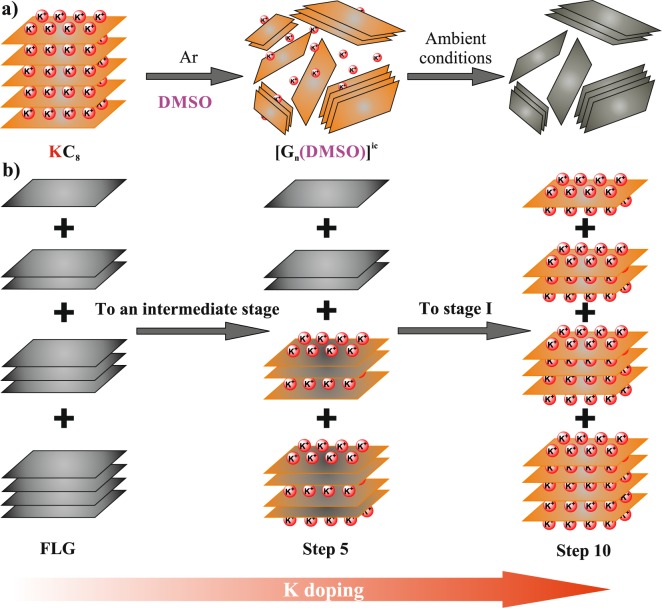
Proposed scheme of alkali doping for the FLG sample. (**a**) Synthesis steps of the starting FLG material. (**b**) Illustration of the *in-situ* intercalation process. The sample is a mixture of a few layers: moderate doping affects the flakes with more layers (Steps 1–7) and higher doping steps (Steps 8–10) results in full doping of all flakes including those consisting of entirely single graphene layers.

Figure 5 summarizes the proposed doping scheme for the FLG sample, which allows to gain insight into the heterogeneous layer number distribution. At the beginning of the K intercalation (Steps 1–7), only a high Stage (Stage 3, as we identified) can be reached, which is geometrically possible only in flakes containing restacked graphene of at least 5 layers. Thus, flakes consisting of less than 5 restacked graphene layers remain intact from potassium doping at these steps. As the doping proceeds, it is only a low amount of flakes that become intercalated, as strictly speaking our doping steps do not form a material in thermodynamical equilibrium due to the inhomogeneous composition. At higher doping (Step 8–10), flakes with smaller thicknesses start to be doped and eventually all graphene layers are doped to saturation, which corresponds to the structure of Stage 1 GIC.

This scenario is supported by first-principles studies^55–57^, i.e., that alkali (potassium^55,56^ or lithium^57^) doping yields a formation energy gain (Δ*F*) that decreases for lower stages up to Stage 2, and increases for Stage 1. This behavior is confirmed by calculations for all thicknesses with a layer number *n* ≥ 3. This staging phenomenon means that all flakes of the sample reach the same stage before a new stage is started to be formed, independently of the layer number. The same effect was found experimentally in bilayer graphene individual flakes, i.e., that the doping occurs first on one of the layers reaching a full Stage 2 doping (top layer, in general) before accumulating in-between all layers^58,59^.

A practical protocol for the use of the present method is as follows. The chemically exfoliated graphene being studied needs to be intercalated with potassium in a stepwise manner along the protocol, which we present in the Methods Section. Clear evidence for a material, which consists of monolayer graphene only, is when the stepwise doping steps result in a fully intercalated material, without the presence of the intermediate stages (e.g. the G mode shifts continuously). When the intermediate steps are present, the typical upper limit of the layer number can be determined in three steps. First, the position of the G_2_ peak has to be compared with that found in GICs. Secondly, the 2D peak has to be fitted to a combination of Lorentzian curves. The position of the lowest fitted Lorentzian curve must be correlated to the highest stage possible in the material.

Note that for samples with flakes with a large fraction of 10 or more layers, our method is only capable to prove that the sample contains multilayer flakes.

Here, we limited ourselves to determine the typical upper limit of the distribution of the number of layers in chemically exfoliated graphene powder samples. However, further development of our analysis of the Raman spectra could yield more information about the precise statistical distribution.

It would be of interest to investigate the stacking of the graphene layers using an extension of our Raman spectroscopy-based technique. Graphene multi-layers typically exhibit two structures of stacking: the more stable AB (Bernal) stacking and the less stable rhombohedral, ABC stacking. The rhombohedral stacking could lead to promising correlated states displaying superconductivity and magnetism^2–4,60,61^. Recently, multilayer graphene flakes with ABC stacking were successfully isolated exceeding 17 graphene layers^62^. This stacking could be identified using Raman spectroscopy as described in recent works^60,62^. Specifically, first-principles calculations demonstrated that the 2D band is broader, and it has several components for ABC stacking. In addition, a shoulder emerges at 2576 cm^−1^ for 1.96 eV laser energy. These Raman fingerprints would be all the more relevant to study due to the interesting arbitrary restacking previously observed in few-layer graphene flakes^63,64^.

## Conclusions

In conclusion, we presented a Raman spectroscopy-based technique to identify the maximal flake thickness in few-layer bulk graphene samples. The presented method is based on studying *in-situ* K doping of FLG samples. Our method uses the combination of the G-band position, its intensity, and the position of the 2D mode components to determine the typically thickest flakes in the sample and to confirm the presence of single-layer ones. The technique works well on FLG powder samples prepared using wet chemical exfoliation technique and was tested for three different mechanical processing routes. Statistical AFM shows that such samples consist of flakes with a non-uniform distribution of the number of graphene layers, and 90% of the flakes consist of mostly less than 5 layers. The Raman spectra of intermediately doped FLG samples can be best described as a sum of two components, corresponding to doped and undoped graphene flakes. The former was argued to arise from five-layer-thick flakes and the latter from flakes made of fewer numbers of graphene layers. Remarkable agreement of our AFM statistical analysis and our Raman data validates our method. Our method provides an efficient way to characterize graphene samples with an arbitrary distribution of the number of graphene layers, where other alternatives (microscopic tools or analysis of the 2D mode) fail.

## Methods

Few-layer graphene samples were prepared from saturation potassium doped SGN18 spherical graphite powder (Future Carbon) using DMSO solvent for the wet chemical exfoliation as described elsewhere^33,65,66^ and as shown in Fig. 5a. Chemical exfoliation was finalized using different mechanical routes: ultrasound sonication, shear mixing and mechanical stirring. In a previous study, we showed that mechanical processing affects the material quality of final product^67^. Further details of the sample preparation are described in Ref. 67.

The as-prepared bulk samples were characterized by atomic force microscopy (AFM) topographic measurements and images to determine the flake size and the typical number of graphene layers in the bulk material. For AFM statistical analysis, the samples were drop-casted on a 100 × 100 *μ*m surface of a Si/SiO_2_ wafer. Unlike the samples used for Raman studies, only partial restacking may occur due to drop-casting and presence of the substrate in this configuration used for AFM studies. These measurements were carried out using a Bruker Dimension Icon microscope in tapping mode. Bruker Scanasyst-Air silicon tips on nitride levers with a spring constant of 0.4 N/m were used to obtain images resolved by 512 × 512 pixels.

To overcome the difficulties of the determination of the individual layer thickness due to residual solvent, capillary forces, and adhesion, we took advantage of the frequently used step-height analysis procedure^68^. We first examined few, incompletely exfoliated nanosheets, which showed clear terraces. As the terrace step heights are multiples of the apparent thickness, we were able to identify the individual layer thickness to be ~1.2 nm.

Potassium intercalation was carried out *in situ* under a vacuum of 4 × 10^−8^ mbar pressure. To perform the *in situ* intercalation process, we used a similar setup to the one presented and described in Fig. 1 of Ref. 59. Prior to doping, the sample was resistively heated up to 500 °C to evaporate the remaining solvent used before. The applied geometry is similar to the two-chamber vapor phase method used for graphite intercalated compounds (GICs)^46^. Due to lack of substrate, significant restacking of individual graphene flakes occurs, leading to misaligned layers in the sample. The powder sample consists of sponge-like structures, as thermodynamic equilibrium is achieved to create a three-dimensional solid material. As a result of the described sample morphology, Raman measurements provide detailed information not only of single graphene flakes but a large ensemble of flakes that are restacked. As restacking creates only mechanical contact, electronic properties of individual flakes are preserved. Thus, the staging phenomenon necessary for our Raman-based technique occurs at the level of individual flakes.

Raman measurements were performed at 458, 514.5, and at 568 nm laser excitation wavelengths. To facilitate our discussion, our manuscript focuses on the observations at 514.5 nm wavelength, only the statistical analysis of the G-band positions in Fig. S4. is presented at all three wavelengths to confirm lack of dispersion. All other observations and our Raman-based method are independent of the wavelength, as well.

A power of 0.5 mW was used to avoid laser-induced deintercalation^49,69^. Potassium with a purity of 99.95% (Sigma-Aldrich MKBL0124V) was evaporated to the sample in several steps in a controlled *in situ* process. At the first step, the potassium was evaporated for about 2 minutes. In the following steps, we used each time twice longer intercalation times than at the previous Step with constant temperature gradient until homogeneous doping was concluded through Raman measurements of the intensity ratio of the G modes (see discussion of Fig. 2). After each stable phase, the intercalation steps were continued with a smaller temperature gradient, and with a starting evaporation time of 2 minutes. This method follows the well-known two-zone vapor phase method^46^. Maximal doping was achieved in approximately 10 steps in all cases. Near saturation doping, a gradual colour change was observed from black to brown and red, respectively. HOPG and single-crystal graphite intercalation compounds show a similar color change, however, no such significant change is apparent for graphite powder samples due to surface roughness^54^. This difference is seen as proof of the smooth surface of the graphene layers in FLG (See supplementary material).

Following each intercalation step, Raman spectra were recorded on a modified broadband LabRAM spectrometer (Horiba Jobin-Yvon Inc.). The built-in interference filter was replaced by a broadband beam splitter plate with 30% reflection and 70% transmission. The principles of the broadband operation are described elsewhere^70,71^. The spectrometer was operated with a 1800 grooves/mm grating. A typical 0.5 mW laser power was used with a built-in microscope (Olympus LMPlan 50 × /0.50 inf./0/NN26.5).

## Supplementary information

Supplementary Material
