# Supplementary material for: Characterizing the maximum number of layers in chemically exfoliated graphene
Source: Sci Rep. 2019 Dec 20;9:19480. doi: 10.1038/s41598-019-55784-6 (PMC6925211; doi:10.1038/s41598-019-55784-6)
Supplement: Supplementary file 1 — Supplementary Material [file 41598_2019_55784_MOESM1_ESM.pdf]

Characterizing the maximum number of layers in chemically exfoliated graphene

P. Szirmai, B. G. Márkus, J. C. Chacón-Torres, P. Eckerlein, K. Edelthalhammer, J. M. Englert, U. Mundloch, A. Hirsch, F. Hauke, B. Náfrádi, L. Forró, C. Kramberger, T. Pichler and F. Simon

SUPPLEMENTARY MATERIAL

Color change upon K doping

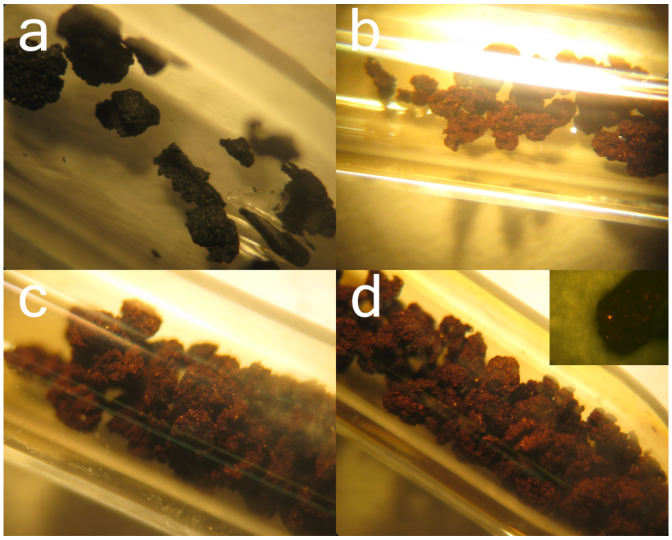

Figure S1. Different doping stages: *a)* undoped material, *b)* after 1 hour doping, *c)* after 1.5 hour doping, *c)* after 2 hour doping, *c)* saturation doped material after 3 hour doping. Inset: Microscopic image of an *in-situ* optimally doped sample.

Figure S1 displays the color of few-layer graphene upon potassium doping. We identified a gradual color change from gray to deep red as shown in Figure S1 (see Figure S2 for another sample). Whereas graphite intercalation compounds show a similar color change (e.g. Stage-1 samples with KC_8 stoichiometry are gold), no such significant change is apparent for graphite powder samples due to surface roughness[1]. This difference is seen as a proof of the smooth surface of the graphene layers in FLG.

Analysis of saturation doped FLG

In Figure S3, an analysis of the saturation doped reference graphite powder (SGN18) and of the chemically exfoliated sample (FLG) is displayed. Both spectra can be well fitted with the sum of an E_{2g2} Breit-Wigner-Fano component, a Lorentzian component around 1560 cm^{-1}, and the C_z mode. As discussed in the main text, the appearance of the Fano line at saturation doping is a signature of the presence of non single-layer species in the powder. The Fano components fitted to the two spectra differ as described in Table 1. of the main text. The weak graphitic component around 1560 cm^{-1} is a common feature with the GICs[2], and it is assigned to the Stage 2 (KC_{24}) level doping in FLG and in SGN18.
Regarding the c-axis mode, the so-called C_z mode around 560 cm^{-1}, it is present in monolayer, few-layer graphene and in KC_8[2,3]. This M-point mode is only Raman active for high intercalation levels. The large intensity of the C_z mode is therefore a manifestation of successful maximal doping in our sample. Whereas the overall intensity in the two samples is comparable, the linewidth and the asymmetry differs in SGN18 and in FLG. The measure of the asymmetry, $|1/q|$ is larger in FLG, pointing to

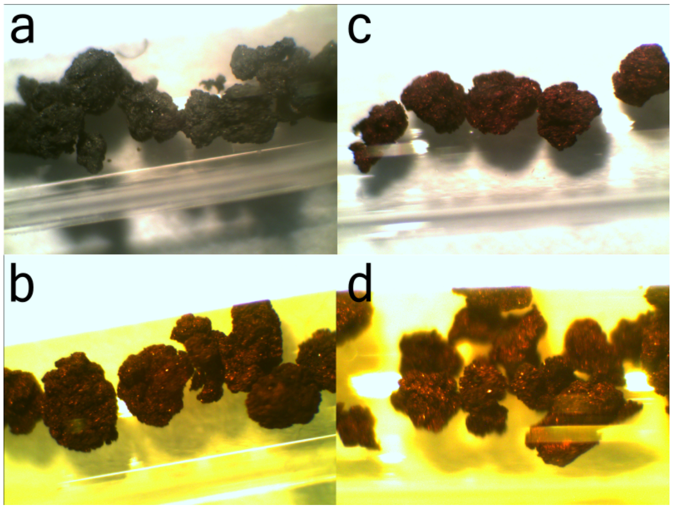

Figure S2. Potassium doped FLG in different doping stages: *a)* undoped material, *b)* after 2.5 hour doping at 200 °C, *c)* after 14.5 hour doping at 200 °C, *c)* after 19 hour doping at 200 °C, *c)* after 37 hour doping at 200 °C and 1.5 hour doping at 300 °C

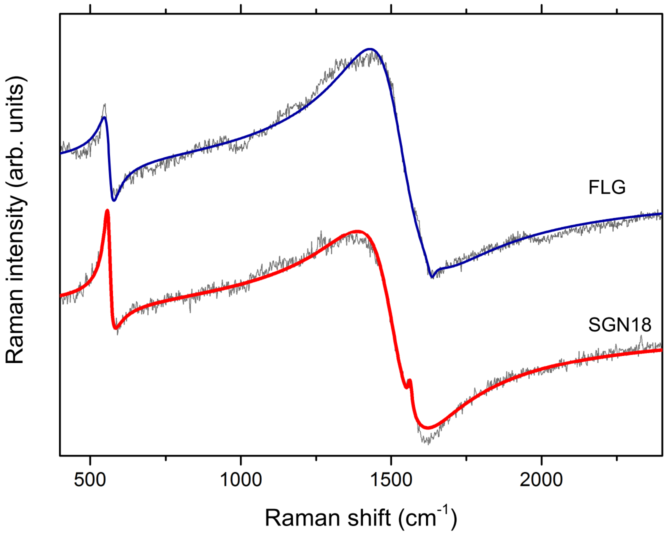

Figure S3. Raman spectra of saturation potassium doped ultrasounded FLG and graphite powder. The fit of the analysis is shown as a thick line.

larger coupling with the electronic continuum. This is plausible as the small number of layer increases the relative doping in FLG. In parallel with this, the broader C_z mode can be assigned to the larger vibration energy uncertainty of the system.

Statistical analysis of the G-mode positions

To compare the G modes of the SGN18 graphite powder and that of the chemically exfoliated FLG, we show in Figure S4 the phonon frequency difference of the G_2 and the G_1 modes ($\Delta\omega_G = \omega(G_2) - \omega(G_1)$) for all doping steps and for three different wavelengths in samples prepared with different mechanical processing routes. In order to facilitate the comparison, the $\Delta\omega_G$ value in KC_{36} is also shown as a dashed line[4]. The Raman shift differences in SGN18 have a minimum at the single crystal value, while it is a maximum for FLG within the error bar of our analysis. Hence, the FLG Raman spectra are, in average, the mixtures of lower G_2 and higher G_1 modes than those found in SGN18. The lower G_2 lines stem from highly charged (close to KC_{36}), the higher strained the higher G_1 lines arise from unstrained few-layer flakes. This is in perfect agreement with our observation of a two-component Raman spectrum and our decomposition of the Raman signal as shown in the main text.

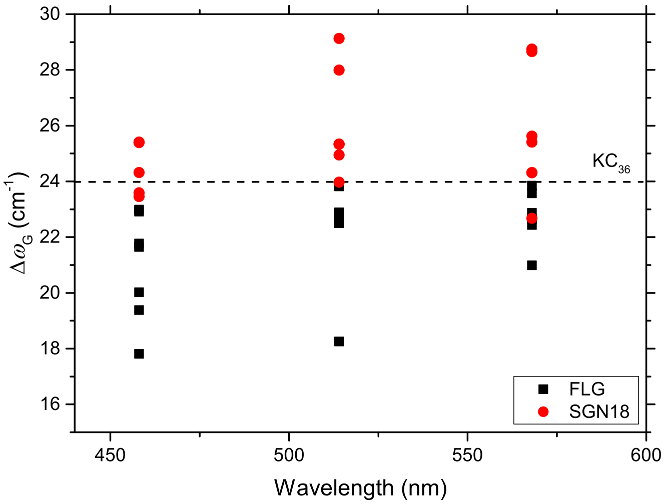

Figure S4. Phonon frequency difference of the G_c and the G_s modes in FLG and in SGN18 at all measured wavelengths and for all doping stages. The dashed line is the Stage 3 single crystal value from Ref.[4].

Intensity ratio of G-mode components

In order to further demonstrate that the Raman spectrum of lightly doped FLG evinces a two-component system, we compare the intensity ratios of the two G lines and the position change of the 2D bands. As we discussed above, the charge transfer happens predominantly from the alkali intercalant to the neighboring graphene layer. This implies that in the GICs, where all the measured sample reaches a constant stage, the intensity ratio of strained and charged G lines (R=Int(G_s)/Int(G_c)) can be correlated with the stage number. In previous works[4,5], a linear increase of R was found as a function of stage number independently of the alkali metal intercalant. Therein, it was shown that it is safe to use the linear dependence of R for the identification of different stages.

In general, this linear correlation reveals that the number of undoped and doped layers, i.e., the K:C ratio can be determined using the R ratio. Our fit to the G lines of lightly doped FLG Raman spectra indicate an intensity ratio of $R \approx 1$ at 514 nm, significantly larger than $R \approx 0.4$ seen in Stage 6 potassium GIC (KC_{72}). Assuming a linear dependence in-between R ratio and the doping level, it evidences an approximate K:C≈1:200 doping level in the first doping step.

In contradiction with the determined doping level, the 2D line exhibits a peculiarly large downshift. As the mode arises from the uncharged inner carbon layers, the 2D line is only present in Stage 3 (KC_{36}) and in higher GIC stages. The downshift of the 2D line components can be derived from the gradually increasing biaxial strain due to lattice expansion upon potassium doping[4]. The downshift by about 40 cm^{-1} found in KC_{36} is notably smaller than the downshift by about 70 cm^{-1} measured in our lightly doped FLG sample.

Thus, the large 2D downshift is in sharp contrast to the K:C=1:200 doping level found for the G modes. Given that the local strain is expected to be smaller in FLG than in single crystal graphite, this discrepancy can only be explained by the above described coexistence of undoped and doped SLG and FLG flakes.

Simulation of FLG Raman spectrum at $\lambda = 633$ nm

Figure S5 shows a similar blow-up at $\lambda = 633$ nm as shown at 514 nm in the main text. (Single crystal Raman spectrum is measured at $\lambda = 647$ nm.) Similarly to the other case, the simulated spectrum (sum of two components) describes well the experimental one. In the Raman spectrum of intermediately doped FLG, the intensity ratio is Int(G_{uc})/Int(G_c)≈ 0.8, similar to the ratio at $\lambda = 514$ nm, whereas it changes significantly in single crystals[4].

Fitting procedure

Individual, symmetric G- and 2D-mode Raman lines were fitted to Lorentzian absorption curves, which were found to be a convenient and reliable method in the case of GICs[4]. Comparison with a more general Voigtian function did not provide any change of the Raman line characteristics. We make use of the weighted sums of Lorentzian curves assuming only a flat

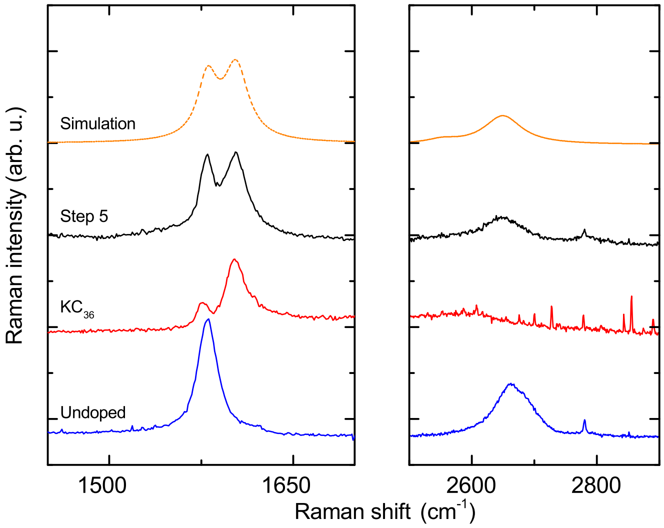

Figure S5. Simulation of intermediately doped FLG Raman spectrum as a superposition of undoped FLG and single crystal Stage 3 Raman spectra at $\lambda = 633$ nm (FLG Raman spectra) and $\lambda = 647$ nm (single crystal Raman spectrum)

background. In general, our fitting curve follows:

$$RS(\nu) = \sum_{i=1}^{n} \mathrm{Int}_i \cdot \left(\frac{1}{\pi} \frac{\frac{1}{2}\Gamma_i}{(\nu - \nu_{0,i})^2 + (\frac{1}{2}\Gamma_i)^2} \right) + \mathrm{Bkg} \tag{1}$$

Here, $RS(\nu)$ is the Raman spectrum to fit as a function of wavenumber, Int_i, Γ_i, and $\nu_{0,i}$ are the intensity (i.e., weight), linewidth, and position of the ith Lorentzian, respectively. Bkg is a constant background.

For fitting Raman spectra, we perform least-square fitting of the experimental data. In other words, given the left-hand side of the equation, we optimize the right-hand side. This is how we fit each Raman spectra in our work.

In addition, we also *simulate* Raman spectra by using fixed values (from the previously fitted values) on the right-hand side to obtain a *simulated* (analytically calculated) Raman spectrum on the left-hand side.

References

1. G. Fábián, B. Dóra, A. Antal, L. Szolnoki, L. Korecz, A. Rockenbauer, N. M. Nemes, L. Forró, F. Simon, Testing the Elliott–Yafet spin-relaxation mechanism in KC_8: A model system of biased graphene, *Phys. Rev. B* **85** 235405 (2012).
2. J. C. Chacón-Torres, A. Y. Ganin, M. J. Rosseinsky, T. Pichler, Raman response of stage-1 graphite intercalation compounds revisited, *Phys. Rev. B* **86** (7) 075406 (2012).
3. C. A. Howard, M. P. M. Dean, F. Withers, Phonons in potassium-doped graphene: The effects of electron-phonon interactions, dimensionality, and adatom ordering, *Phys. Rev. B* **84** 241404(R) (2011).
4. J. C. Chacón-Torres, L. Wirtz, T. Pichler, Manifestation of charged and strained graphene layers in the Raman response of graphite intercalation compounds, *ACS Nano* **7** (10) 9249–9259 (2013).
5. S. Solin, Raman and IR studies of graphite intercalates, *Physica B+C* **99** (1-4) 443–452 (1980).
